# Reassessment of the capacity of the HIV-1 Env cytoplasmic domain to trigger NF-κB activation

**DOI:** 10.1186/s12985-018-0941-7

**Published:** 2018-02-17

**Authors:** Cyprien Beraud, Morgane Lemaire, Danielle Perez Bercoff

**Affiliations:** Department of Infection and Immunity, Molecular Signaling and Virus-Host Interactions group, Luxembourg Institute of Health, 29, rue Henri Koch, L-4354 Esch-sur-Alzette, Luxembourg

**Keywords:** HIV-1, Env cytoplasmic domain, NF-κB, Transcription

## Abstract

The cytoplasmic domain of lentiviral Envelopes (EnvCD) ensures Env incorporation into nascent virions and regulates Env trafficking to and from the plasma membrane. It has also been reported to promote transcription from the viral LTR both directly and indirectly. Noticeably, the HIV-1 and SIV_mac239_ EnvCDs were described to trigger nuclear translocation of NF-κB (Postler, Cell Host Microbes 2012). Given the paramount importance of identifying viral and host factors regulating HIV transcription, cellular signaling pathways and latency, and given that viral replication capacity is dependent on Env, we asked whether HIV EnvCDs from different HIV-1 subtypes differently modulated NF-κB. To that aim, we evaluated the ability of primary HIV-1 Envs from subtypes B and C to activate the NF-κB pathway. Primary subtype B and C Envs all failed to activate the NF-κB pathway. In contrast, when the EnvCD of HIV-1 Envs was fused to the the CD8-α chain, it induced ~ 10-fold increase in NF-κB induction, and this increase was much stronger with a truncated form of the HIV EnvCD lacking the 76 C-terminal residues and containing the proposed TAK-1 binding domain. Our results indicate that the HIV-1 EnvCD is unlikely to trigger the NF-κB pathway in its native trimeric form.

**Electronic supplementary material:**

The online version of this article (10.1186/s12985-018-0941-7) contains supplementary material, which is available to authorized users.

## Manuscript

The cytoplasmic domain (CD) of lentiviral envelopes (Env) is unusually long (~ 150 residues) compared to other retroviruses (< 50 residues) [1] and reviewed in [2–5]. It comprises a disordered sequence with a tyrosine-based internalization signal immediately downstream of the membrane-spanning-domain (MSD), an immunodominant epitope and three amphipathic α-helices (lentiviral lytic peptides, LLP-2, LLP-3 and LLP-1). Despite considerable sequence variation, the physicochemical and structural properties of peptides spanning the LLP regions are believed to be conserved across HIV types and subtypes [6].

The EnvCD ensures Env incorporation into the nascent virion [7–16]. It also regulates Env trafficking to and from the plasma membrane [17, 18] through the endolysosomal and Trans-Golgi-Network (TGN) by interacting with multiple cellular factors, including AP1–3, TIP47, Rab9, Rab11A/FIP1C and retromer components Vps26 and Vps35 [19–25]. Different groups have reported that the EnvCD could also enhance viral transcription, by relieving RhoA-mediated transcriptional inhibition through the interaction of LLP-3 with p155-RhoGEF [26, 27] and by affecting the stability of the precursor of luman, a repressor of Tat-mediated HIV transcription [28]. The HIV-1 and SIV_mac239_ EnvCDs were also reported to induce the nuclear translocation of NF-κB p65/RelA [29]. For HIV-1, residues 759–770, encompassing the Y_768_HRL motif at the N-terminus of LLP-2 interact with TAK-1, leading to phosphorylation of IκB [29].

In vitro, differences in viral replication capacity across subtypes map to the viral Env [30–34]. Because NF-κB activates T-lymphocytes and the viral promoter LTR contains NF-κB binding sites [35], we asked whether primary HIV-1 Envs from subtypes B and C differently trigger the NF-κB pathway.

**Fig. 1:**
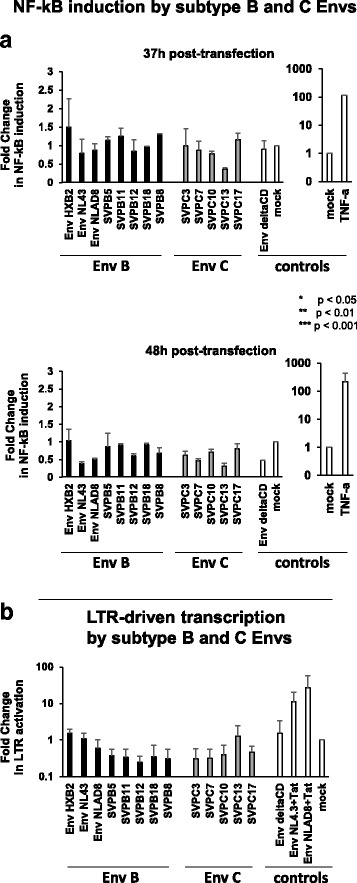
The HIV-1 and SIV CDs do not trigger NF-κB activation. **a** Induction of NF-κB by a panel of HIV-1 subtype B and C Envs. 1.2 × 10^5^ HEK293T cells were cotransfected in duplicate wells with 500 ng of pcDNA-Env expressing vector, 200 ng of NF-κB-Firefly-Luciferase vector and 50 ng of pGL4.74-Renilla-Luciferase for normalization using the Calcium Phosphate precipitation method. We used a panel of full-length Envs cloned in pCDNA3.1: Env of pNL4.3, Env of pNLAD8, 5 primary subtype B Envs (Env_SVPB5_, Env_SVPB11_, Env_SVPB12_, Env_SVPB18_, Env_SVPB8_), 5 primary subtype C Envs (Env_SVPC3_, Env_SVPC7_, Env_SVPC10_, Env_SVPC13_, Env_SVPC17_) and EnvΔCD as negative control. All Env vectors express the two Rev exons. As a positive control, NF-κB was triggered with 100 ng/ml TNF-α 31 or 42 h post-transfection. After 37 and 48 h, Firefly- and Renilla-Luciferase were measured in cell lysates using the Dual-Glo Luciferase kit (Promega) and the Firefly-Luciferase signal was normalized using the Renilla-Luciferase. Results are expressed as Fold-Change in NF-κB induction with respect to the empty pcDNA3.1 vector (mock). The mean of at least two independent experiments is reported. Error bars represent standard error. **b** Induction of transcription from the viral LTR by HIV-1 subtype B and C Envs. 8 × 10^4^ TZM-bl cells were cotransfected with 1 μg of pcDNA-Env expressing vector and 100 ng pGL4.74-Renilla-Luciferase in duplicate wells. LTR-driven transcription (Firefly-Luciferase) was assessed in cell lysates after 48 h (no signal was detected 37 h post-transfection) and normalized using the Renilla-Luciferase. As a positive control, Env expression vectors containing Tat were used. The empty pcDNA3.1 vector (mock) was used for standardization. The mean of three independent experiments is reported. Error bars represent standard error. Statistical analyses for **a** and **b** were performed with GraphPad Prism (version 5). NF-κB induction (**a**) and LTR activation (**b**) were compared using a Kruskal-Wallis test followed by a Dunn’s post-test and differences were considered significant if *p* < 0.05

To evaluate NF-κB induction by different primary HIV-1 Envs, HEK293T cells were cotransfected with a NF-κB-Firefly-Luciferase reporter plasmid and a panel of 13 HIV-1 full-length Envs cloned in pCDNA3.1: we used HIV-1 Env_NL4.3_ [36], Env_NLAD8_ [37], Env_HXB2_, subtype B [38] and subtype C [39, 40] Envs. Env_NL4.3_ harboring a STOP codon at position 710 (EnvΔCD) was used as negative control. All vectors express the two Rev exons. Transfection efficiency was assessed by Flow Cytometry and confirmed protein expression 37 and 48 h post-transfection, with a decrease by 48 h post-transfection (Additional file 1: Figure S1A), probably reflecting Env-induced cell death. To normalize for transfection efficiency, a plasmid expressing CMV-Renilla-Luciferase (Promega pGL4.74 hRLuc) was included in all experiments. NF-κB-Luciferase induction by each of the viral Envs was normalized using the corresponding Renilla-Luciferase signal, and calculated as the fold-change relative to the empty vector (mock), as in [29]. As shown in Fig. 1a, TNF-α (Sigma) readily induced a ~ 2 log increase in NF-κB-Luciferase, validating the system. However, neither of the HIV-1 Envs triggered NF-κB activity (*p* > 0.05, Kruskal-Wallis test): NF-κB-Luciferase induction ranged from 0.79 to 1.5 for subtype B Envs and from 0.36 to 1.16 for subtype C Envs 37 h post-transfection and from 0.36 to 1.04 for subtype B Envs and 0.31 to 0.80 for subtype C Envs 48 h post-transfection. Variations in NF-κB-Luciferase (Fig. 1a) did not recapitulate Env expression levels (Additional file 1: Figure S1A). When NF-κB-Luciferase induction was further normalized to Env expression levels (MFI) to account for variability in Env expression levels, NF-κB-Luciferase triggered by the viral Envs never exceeded the levels induced by the mock control (*p* > 0.05, Kruskal-Wallis test) (Additional file 2: Figure S2A), reflecting basal cell activation levels upon transfection and confirming that native Envs do not trigger NF-κB. Limiting serum in HEK293T cell cultures (1% Fetal Bovine Serum) to ensure minimal basal activation did not change NF-κB induction (not shown). Of note, while the HIV-1 Env ectodomain has been reported to trigger NF-κB and apoptosis [41–43], this phenomenon requires CD4 and CXCR4 or a co-receptor. Here we investigated NF-κB-induction in cells that do not express the viral receptor CD4, excluding a similar phenomenon. The capacity of the HIV-1 Envs to induce transcription from the LTR was then assessed by transfecting TZM-bl cells with the same Env expression vectors. TZM-bl cells are CD4^+^ CXCR4^+^ HeLa-derived cells expressing the Firefly Luciferase and the β-galactosidase genes under the control of the viral promoter LTR. Tat-containing Env expression vectors (Env_NL4.3 + Tat_, Env_NLAD8 + Tat_) were used as positive controls and the CMV-Renilla-Luciferase vector was included for normalization. LTR-driven transcription was induced by the Tat-containing vectors, as expected, but not by the Env expression vectors, ranging from 0.25 to 1.51 and from 0.30 to 1.26 for subtype B and C Envs respectively (*p* > 0.05, Kruskal-Wallis test) (Fig. 1b).

**Fig. 2:**
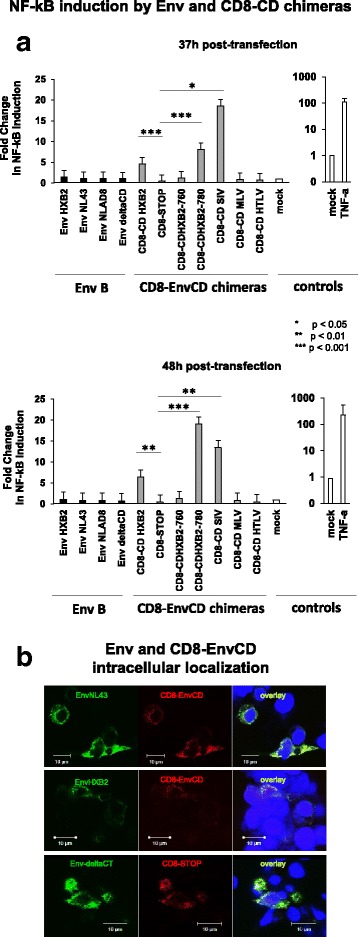
The EnvCD activates NF-κB when fused to the CD8-α chain. **a** Comparison of the ability of native Env and CD8-EnvCD chimeras to activate NF-κB. 1.2 × 10^5^ HEK293T cells were cotransfected with 200 ng of NF-κB-Firefly-Luciferase vector, 50 ng of pGL4-Renilla-Luciferase and 500 ng of pcDNA-Env expressing vectors (Env_HXB2_, Env_NL4.3_, Env_NLAD8_, EnvΔCD) or the following CD8-EnvCD chimeric constructs: CD8-EnvCD_HXB2_ (residues 707–856 of Env_HXB2_), CD8_STOP_, CD8-EnvCD_HXB2Δ3_ (residues 707–760 of Env_HXB2_), CD8-EnvCD_HXB2Δ4_ (residues 707–780 of CD_HXB2_), CD8-EnvCD_SIVmac239_ (residues 716–879 of Env_SIVmac239_), CD8-EnvCD_MLV_ (residues 640–665 of Env_MLV_) and CD8-EnvCD_HTLV-I_ (residues 466–488 of Env_HTLV-I_). Transfections were performed in duplicate wells. Firefly and Renilla-Luciferase activities were recorded 37 and 48 h post-transfection. The Firefly-Luciferase signal was normalized to the Renilla-Luciferase signal. The empty pcDNA3.1 vector was used as negative control (mock) and was used for standardization. The mean of seven independent experiments is reported. Error bars represent standard error. NF-κB activation by different constructs was compared by a Kruskal-Wallis test followed by a Dunn’s post-test using Graph Pad Prism version 5.0 and differences were considered significant if *p* < 0.05. **b** Intracellular localization of EnvNL43, EnvHXB2 and CD8-EnvCD. 1.2 × 10^5^ HEK293T cells were cotransfected with 200 ng of Env_NL4.3_ or Env_HXB2_ and CD8-EnvCD or with EnvΔCD and CD8_STOP_. After 48 h, cells were washed and fixed with cold absolute ethanol and stained with a polyclonal goat α-Env antibody (Abcam ab53937) and Rabbit anti-CD8α antibody (H-160, Santa Cruz), then sequentially incubated with donkey anti-goat IgG then goat anti-mouse and anti-Rabbit IgG secondary antibodies coupled to Alexa Fluor 488 and Alexa Fluor 568 (Invitrogen). Images were captured with a Zeiss LSM510 META confocal laser scanning microscope (Jena, Germany) equipped with a 63× Plan-NeoFluar oil immersion objective (numerical aperture 1.3)

One major difference between our experimental set-up and that of Postler et al. [29] lies in the use of Env expression vectors versus CD8-EnvCD chimeras, respectively. To verify the impact of the ectodomain on the ability of the EnvCD to trigger the NF-κB pathway, we cotransfected HEK293T cells with the NF-κB-Luciferase reporter and a construct containing the EnvCD of HXB2 (residues 707–756) fused to the extracellular and transmembrane domains of the CD8-α chain (residues 1–211) [20], a kind gift from C Berlioz-Torrent. A CD8-α construct bearing a STOP codon downstream of the transmembrane domain (CD8_STOP_) was used as a negative control [20]. The CMV-Renilla-Luciferase vector was included for normalization and the fold-change in NF-κB-Luciferase induction was compared (Kruskal-Wallis test). As expected, the CD8-EnvCD_HXB2_ chimera induced a ~ 10-fold increase in NF-κB-dependent-Luciferase expression relative to the CD8_STOP_ construct 37 h (*p* < 0.001) and 48 h (*p* < 0.01) post-transfection (Fig. 2a), in agreement with the findings of Postler et al. using a similar chimera [29]. Using a CD8-EnvCD chimera truncated just downstream of the Y_768_HRL motif, CD8-EnvCD_HXB2–780_ (residues 707–780 of HIV-1 EnvCD_HXB2_), NF-κB-Luciferase activity was ~ 16-fold and ~ 40-fold higher relative to CD8_STOP_ 37 and 48 h post-transfection, respectively (*p* < 0.001) (Fig. 2a), while a CD8-EnvCD chimera truncated just upstream of the motif of interest, CD8-EnvCD_HXB2–760_ (residues 707–760 of HIV-1 EnvCD_HXB2_) did not activate the NF-κB pathway (Fig. 2a), again recapitulating the results of Postler et al. using a CD8-EnvCD construct lacking the 74 C-terminal residues [29]. When NF-κB induction was further normalized to CD8-EnvCD expression levels, CD8-CD_HXB2_, CD8-EnvCD_780_ and CD8-SIV_mac239_ maintained the capacity to activate NF-κB compared to the CD8_STOP_ construct (Additional file 2: Figure S2B). Taken together, these results show that the HIV-1 EnvCD triggers the NF-κB pathway only when expressed downstream of CD8-α, but not in its wild-type form downstream of the isogenic Env ectodomain. We then verified the intracellular localization of the Env-based and CD8-based constructs. As shown in Fig. 2b, Env_NL4.3_ and Env_HXB2_ colocalized nicely with CD8-EnvCD_HXB2_ and EnvΔCD colocalized with CD8_STOP_, arguing against the possibility that different intracellular localization accounts for this dichotomy. We also evaluated the ability of CD8-α-based chimeras fused to the EnvCDs of SIV_mac239_, MLV and HTLV-1 fused to the CD8-α chain [20] to trigger NF-κB. The CD8-EnvCD_SIVmac239_ induced a ~ 26-fold (*p* < 0.05) and 36-fold (*p* < 0.01) increase in NF-κB-Luciferase 37 and 48 h post-transfection, respectively, compared to CD8_STOP_ (Fig. 2a). The short EnvCDs of MLV and HTLV had no impact on NF-κB activity (*p* > 0.05) (Fig. 2a), probably because they lack LLP domains. NF-κB induction by CD8-EnvCD_HXB2_ and CD8-EnvCD_HXB2–780_ was higher 48 h post-transfection than 37 h post-transfection, while NF-κB induction by CD8-EnvCD_SIVmac239_ was weaker 48 h post-transfection, probably reflecting EnvCD_SIVmac239_ toxicity.

Given that T lymphocyte activation is a prerequisite to HIV replication and that the viral promoter LTR contains NF-κB binding sites, identifying the factors that do promote viral transcription and induce apoptosis in a physiological setting is of major importance. It has been proposed that together with Nef, the EnvCD could provide CD4^+^ T-lymphocytes the two independent triggers necessary for cell activation and viral replication in vivo. Our results clearly argue against the possibility that the HIV-1 EnvCD might trigger the NF-κB pathway during HIV-1 infection*.* One possible explanation to the differences observed using CD8-EnvCD chimeras and full length HIV-1 Envs is that differences in conformational dynamics dictate the ability of the HIV-1 EnvCD to trigger the NF-κB pathway. Determinants involved in NF-κB induction might remain cryptic in the trimeric native form of Env while becoming exposed in the context of CD8-EnvCD chimeras. The N-terminal domain of the constructs (Env-ectodomain or CD8-α) may affect the conformation of the EnvCD. The reverse has been reported in that truncations of the HIV-1 or SIV_mac239_ EnvCDs affect the conformation of the corresponding extracellular domain and its susceptibility to neutralization [44, 45]. The levels of Env oligomerization may further modify the determinants of Env which are exposed. In the CD8-EnvCD chimeras, the EnvCD is most likely mono- or dimeric given that CD8 is dimeric [46]. In the native Env, the EnvCD is mainly trimeric. These possibilities are in line with the observation that truncated forms of the EnvCD are more potent NF-κB pathway activators than the full-length Env. While CD8-α-based chimeras and truncated proteins are powerful tools to dissect the biochemical properties and molecular interactions of retroviral EnvCDs, they have limitations, including potential conformational discrepancies with the native protein, as this study documents, and the fact that truncated EnvCDs are counter-selected in vivo for Env incorporation is impaired [47]. Further studies will be needed to fully appreciate the structure and functions of the HIV-1 EnvCD.

## Conclusions

In conclusion, the EnvCD of HIV-1 seems to trigger NF-κB when expressed downstream of CD8-α, particularly when truncated forms of the EnvCD are used, but this effect does not extend to the native Env, arguing against the likelihood that the HIV EnvCD activates this pathway in its native form. The results reported in this study confirm the crucial role of the native trimeric structure of the HIV-1 Env protein and illustrate the need to interpret data obtained with chimeric constructs with the highest caution, first ensuring they extend to native proteins. Given that the viral Env is the target of neutralizing antibodies and given the chief role of cellular activation in the pathogenesis of HIV-AIDS, accurately identifying epitopes with potential biological functions is of major importance for the understanding of HIV pathology and for the design of protective vaccine and viral reservoir eradication strategies.

## Additional files

Additional file 1: Figure S1. Expression of Env and CD8-EnvCD 37 and 48 h post-transfection by Flow Cytometry. A. Expression of subtype B and C Env in HEK293T cells. 1.2 × 10^5^ HEK293T cells in duplicate wells were cotransfected in the same conditions as in Fig. 1a with all Env expression vectors and the Luciferase expression vectors. The empty pcDNA3.1 vector was used as negative control (mock). Duplicate wells were pooled and Env expression was measured by flow cytometry 37 and 48 h post transfection using a 1:1 mixture of human anti-gp120 antibodies PGT121 + F105 (AIDS Research and Reagent program) and an APC-labelled mouse anti-human IgG secondary antibody (Lifetech A21445). Analyses were performed using FlowJo v10. The mean MFI of at least 3 independent experiments are reported. Error bars represent standard deviation. B. Expression of reference Env and CD8-EnvCD chimeras in HEK293T cells. 1.2 × 10^5^ HEK293T cells in duplicate wells were cotransfected in the same conditions as in Fig. 2a with Env and CD8-EnvCD expression vectors and the luciferase expressing vectors. The empty pcDNA3.1 vector was used as negative control (mock). Duplicate wells were pooled and cells were stained either with the same 1:1 mixture of human anti-gp120 antibodies PGT121 + F105 and an APC-labelled mouse anti-human IgG secondary antibody or with a 510-labelled mouse anti-human CD8 antibody (Biolegend #301048). Analyses were performed using FlowJo v10. The mean MFI of at least 3 independent experiments are reported. Error bars represent standard deviation. (PDF 302 kb)

Additional file 2: Figure S2. NF-κB induction relative to Env and CD8-EnvCD expression levels. A. NF-κB induction by subtype B and subtype C Envs relative to Env expression levels. NF-κB induction measured in HEK cells co-transfected with the subtype B or subtype C Envs, NF-κB-Luciferase and CMV-Renilla-Luciferase vectors (Fig. 1a and b) was normalized to Env expression levels (MFI, Additional file 1: Figure S1A) to account for differences in Env expression vectors. B. NF-κB induction by CD8-EnvCD relative to expression levels. NF-κB induction measured in HEK cells co-transfected with the CD8-EnvCD constructs, NF-κB-Luciferase and CMV-Renilla-Luciferase vectors (Fig. 2a and b) was normalized to CD8-EnvCD expression levels (MFI, Additional file 1: Figure S1B) to account for differences in expression vectors. It is noteworthy that this second normalization round is subject to differences in antibody affinity for Env, in Env expression kinetics and cycling dynamics, as well as in Env-induced cytotoxicity. This is particularly the case for the subtype B and C primary Envs, while CD8-EnvCD expression levels are less subject to differences in antibody affinity. (PDF 308 kb)

## Abbreviations

AP: Adaptor Protein
Env: Envelope
EnvCD: Envelope Cytoplasmic domain
HIV-1: Human Immunodeficiency Virus type 1
HTLV-I: Human T-cell Leukemia virus type I
LLP: Lentiviral Lytic Peptide
MLV: Murine Leukemia Virus
MSD: Membrane Spanning Domain
SIV: Simian Immunodeficiency Virus
