# Supplementary material for: Reassessment of the capacity of the HIV-1 Env cytoplasmic domain to trigger NF-κB activation
Source: Virol J. 2018 Feb 17;15:35. doi: 10.1186/s12985-018-0941-7 (PMC5816530; doi:10.1186/s12985-018-0941-7)

Supplementary data: Env and CD8-CD chimera expression (MFI)

Subtype B and C Env expression

A

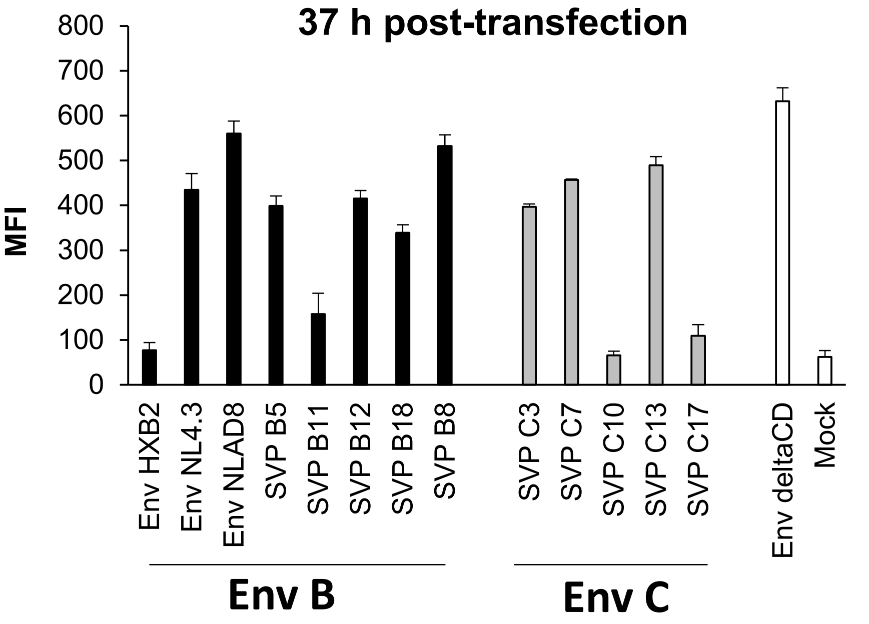

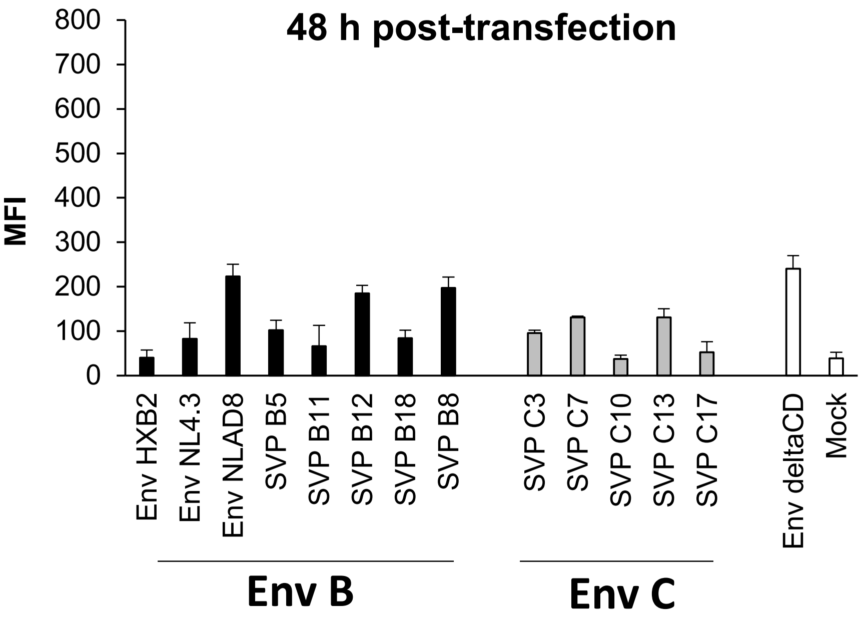

Env and CD8-EnvCD expression (MFI)

B

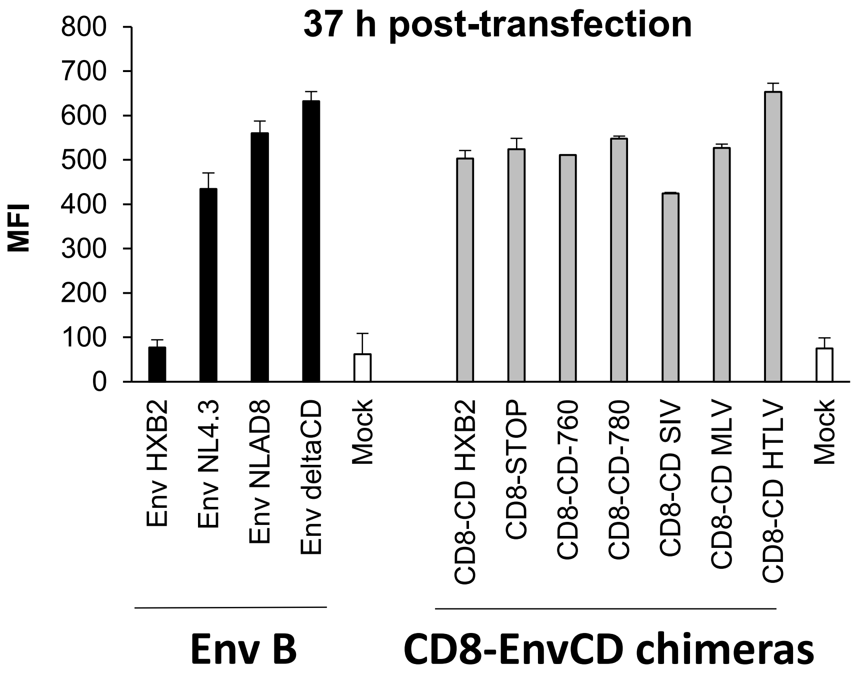

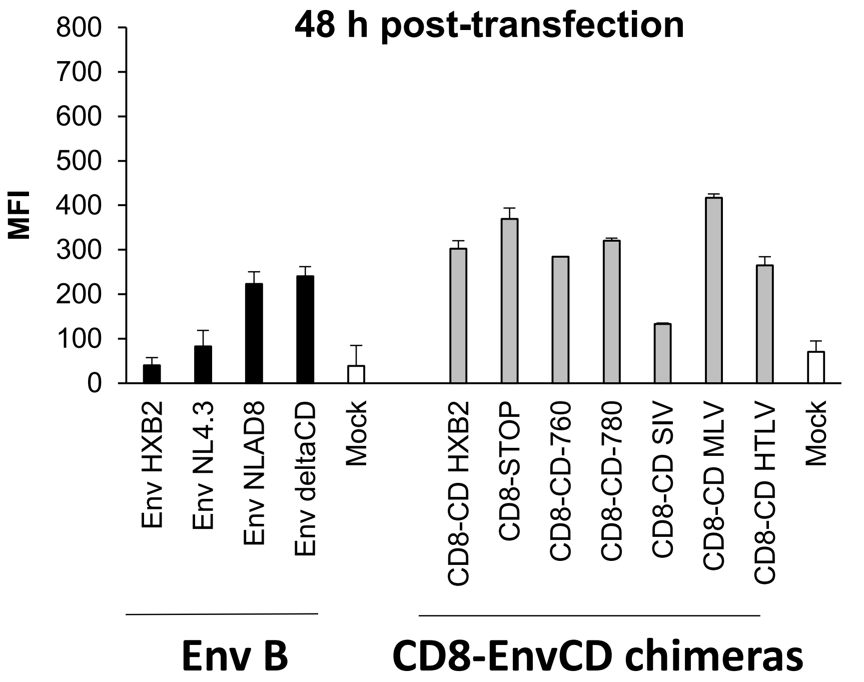

Supplement: Supplementary file 1 — Expression of Env and CD8-EnvCD 37 and 48 h post-transfection by Flow Cytometry. A. Expression of subtype B and C Env in HEK293T cells. 1.2 × 10^5^ HEK293T cells in duplicate wells were cotransfected in the same conditions as in Fig. 1a with all Env expression vectors and the Luciferase expression vectors. The empty pcDNA3.1 vector was used as negative control (mock). Duplicate wells were pooled and Env expression was measured by flow cytometry 37 and 48 h post transfection using a 1:1 mixture of human anti-gp120 antibodies PGT121 + F105 (AIDS Research and Reagent program) and an APC-labelled mouse anti-human IgG secondary antibody (Lifetech A21445). Analyses were performed using FlowJo v10. The mean MFI of at least 3 independent experiments are reported. Error bars represent standard deviation. B. Expression of reference Env and CD8-EnvCD chimeras in HEK293T cells. 1.2 × 10^5^ HEK293T cells in duplicate wells were cotransfected in the same conditions as in Fig. 2a with Env and CD8-EnvCD expression vectors and the luciferase expressing vectors. The empty pcDNA3.1 vector was used as negative control (mock). Duplicate wells were pooled and cells were stained either with the same 1:1 mixture of human anti-gp120 antibodies PGT121 + F105 and an APC-labelled mouse anti-human IgG secondary antibody or with a 510-labelled mouse anti-human CD8 antibody (Biolegend #301048). Analyses were performed using FlowJo v10. The mean MFI of at least 3 independent experiments are reported. Error bars represent standard deviation. (PDF 302 kb) [file 12985_2018_941_MOESM1_ESM.pdf]
