# Supplementary material for: Reassessment of the capacity of the HIV-1 Env cytoplasmic domain to trigger NF-κB activation
Source: Virol J. 2018 Feb 17;15:35. doi: 10.1186/s12985-018-0941-7 (PMC5816530; doi:10.1186/s12985-018-0941-7)

NF-κB induction by Subtype B and C Env relative to Env expression (MFI)

A

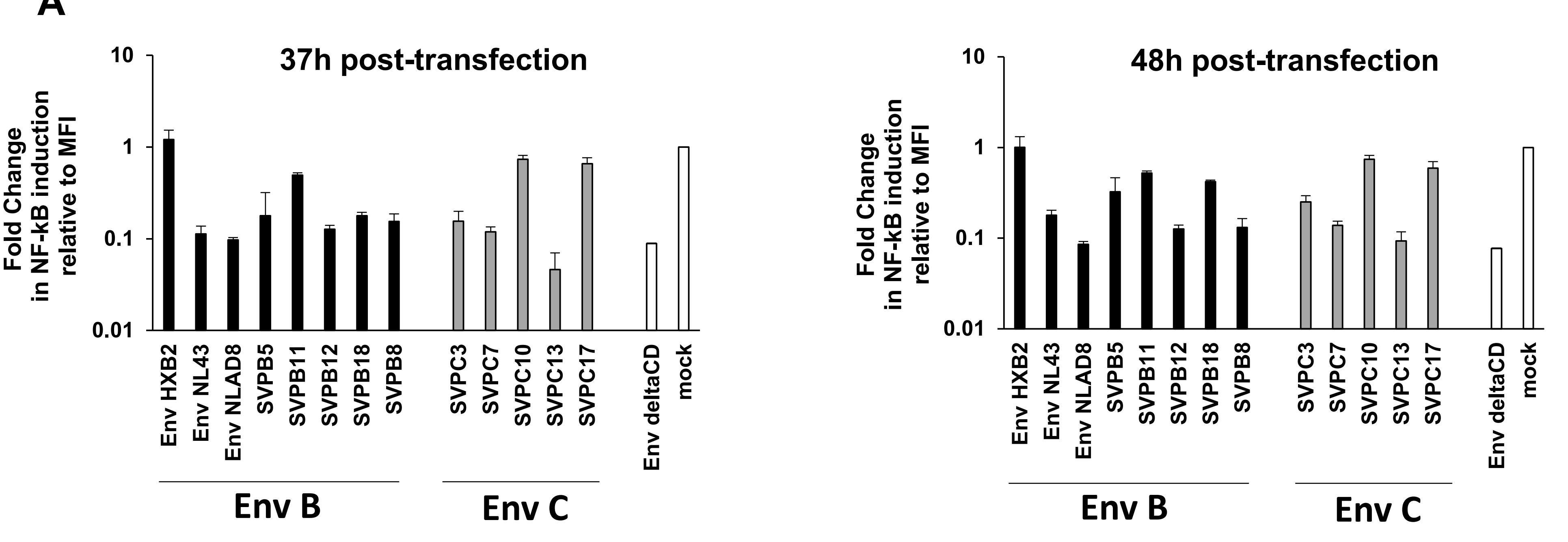

NF-κB induction by CD8-EnvCD chimeras relative to expression levels (MFI)

B

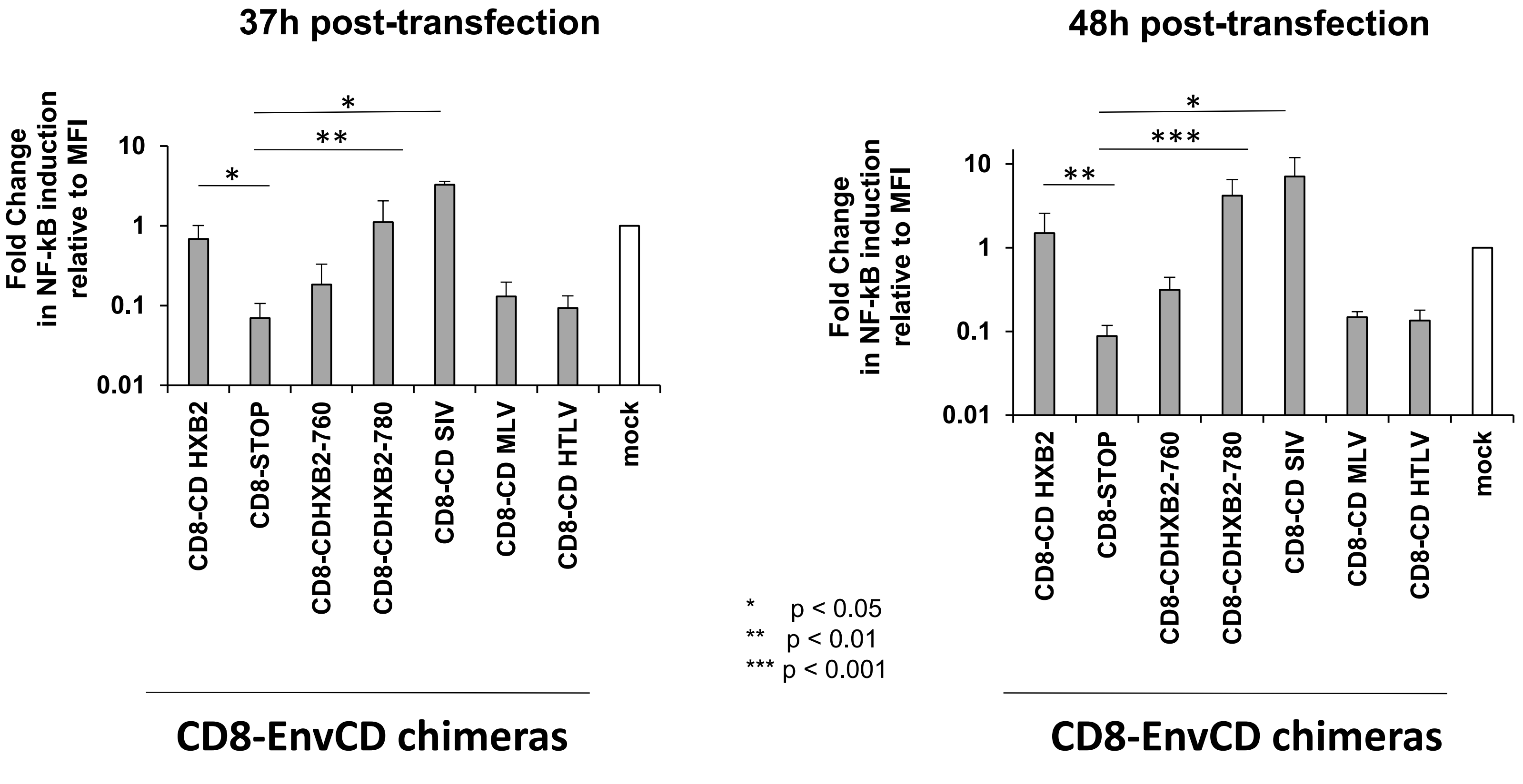

Supplement: Supplementary file 2 — NF-κB induction relative to Env and CD8-EnvCD expression levels. A. NF-κB induction by subtype B and subtype C Envs relative to Env expression levels. NF-κB induction measured in HEK cells co-transfected with the subtype B or subtype C Envs, NF-κB-Luciferase and CMV-Renilla-Luciferase vectors (Fig. 1a and b) was normalized to Env expression levels (MFI, Additional file 1: Figure S1A) to account for differences in Env expression vectors. B. NF-κB induction by CD8-EnvCD relative to expression levels. NF-κB induction measured in HEK cells co-transfected with the CD8-EnvCD constructs, NF-κB-Luciferase and CMV-Renilla-Luciferase vectors (Fig. 2a and b) was normalized to CD8-EnvCD expression levels (MFI, Additional file 1: Figure S1B) to account for differences in expression vectors. It is noteworthy that this second normalization round is subject to differences in antibody affinity for Env, in Env expression kinetics and cycling dynamics, as well as in Env-induced cytotoxicity. This is particularly the case for the subtype B and C primary Envs, while CD8-EnvCD expression levels are less subject to differences in antibody affinity. (PDF 308 kb) [file 12985_2018_941_MOESM2_ESM.pdf]
